# Age and Not the Preferred Limb Influences the Kinematic Structure of Pointing Movements

**DOI:** 10.3390/jfmk6040100

**Published:** 2021-12-08

**Authors:** Kurt W. Kornatz, Brach Poston, George E. Stelmach

**Affiliations:** 1 Department of Exercise Physiology, Winston-Salem State University, Winston-Salem, NC 27110, USA; kornatzkw@wssu.edu; 2 Department of Kinesiology and Nutrition Sciences, University of Nevada-Las Vegas, Las Vegas, NV 89154, USA; 3 Department of Kinesiology, Arizona State University, Tempe, AZ 85281, USA; stelmach@asu.edu

**Keywords:** aging, laterality, aiming, submovement

## Abstract

In goal-directed movements, effective open-loop control reduces the need for feedback-based corrective submovements. The purpose of this study was to determine the influence of hand preference and aging on submovements during single- and two-joint pointing movements. A total of 12 young and 12 older right-handed participants performed pointing movements that involved either elbow extension or a combination of elbow extension and horizontal shoulder flexion with their right and left arms to a target. Kinematics were used to separate the movements into their primary and secondary submovements. The older adults exhibited slower movements, used secondary submovements more often, and produced relatively shorter primary submovements. However, there were no interlimb differences for either age group or for the single- and two-joint movements. These findings indicate that open-loop control is similar between arms but compromised in older compared to younger adults.

## 1. Introduction

The preferential use of one hand over the other is seen in most activities of daily living, with the majority of the population preferring to use the right hand to perform skilled tasks such as writing or manipulating objects [1]. This preferential hand use has a long history and has been attributed to biological, evolutionary, sociological, and environmental factors, which all may contribute to the performance differences between the hands that are often expressed. For example, the dominant hand is quicker and less variable in finger tapping and sequencing tasks [2,3]. In addition, the dominant upper limb (i.e., arm and hand) often expresses less error in the initial impulse [4], fewer corrective adjustments in the trajectory [5], and especially more efficient inter-segmental coordination [6,7] in reaching movements. However, the dominant limb does not always perform better than the non-dominant limb. The non-dominant limb has been shown to be superior at tasks requiring the stabilization of position such as securely holding an object [8,9]. This implies that limbs are functionally specialized with complementary but differing control processes, which is the basis of the dynamic-dominance hypothesis of handedness [8,10,11].

Brain imaging and electrophysiological techniques have provided additional support for differences underlying the neural processes involved in the control of the dominant and non-dominant limbs. Anatomical and functional MRI, transcranial magnetic stimulation (TMS) of the motor cortex, and peripheral nerve stimulation have been used to detect differences in cortical motor representation and corticospinal pathway function for dominant and non-dominant limb movements [12,13,14,15]. Biomechanical descriptions of movements have also been used extensively for examining differences in the activation strategy used by the nervous system to produce movements. For example, Sainburg and colleagues provided clear evidence of control differences in studies that examined kinematics and kinetics of arm movements [6,7,8,9]. Another way to biomechanically examine control strategies for reaching, aiming, and pointing movements is by partitioning the movements into components (i.e., submovements) based upon their velocity, acceleration, and jerk records [16,17]. Generally, if the accuracy requirements are small (i.e., low index of difficulty), a movement to a target will comprise only an initial impulse and exhibit a smooth bell-shaped velocity record. However, if the accuracy demands are large (i.e., high index of difficulty), one or more corrective secondary movements will be used to accurately achieve the target [18,19]. Secondary submovements are distinct modifications to the movement trajectory and are identified by irregularities or fluctuations in the velocity records occurring after peak velocity. The initial impulse or primary submovement is considered to be mediated by open-loop control processes [20] whereas secondary submovements have been considered as a result of feedback, obtained while the movement is in progress, used to achieve the target accurately [4,16]. This is notable, as a number of authors have suggested that the dominant limb system is more adept at the use of open-loop control and the non-dominant limb system more efficient at utilizing feedback in a closed-loop control manner [21,22,23], although these findings have not been proven conclusively [24,25].

Older adults generally exhibit reductions in strength and force control that can lead to diminished functional capabilities [26]. The diminished movement capabilities have been attributed to changes in the neuromuscular system including changes in the number and size of motor units [27,28,29], adaptations in the biophysical and discharge properties of motor neurons, and a decline in sensory capabilities [30]. These physiological adaptations accompany changes in the kinematic structure of movements in healthy older adults [31]. For example, in arm and hand movements, it has been shown that there is a reduction in the duration of the primary submovement and an increase in the number of secondary submovements used to achieve the target [18,32]. As there is a relation between movement complexity and accuracy, it is likely that the submovement structure would be further exacerbated by the performance of multi-joint movements, especially for older adults, due to the increased complexity of movement planning and execution [33,34,35].

The purpose of this study was to compare the kinematic structure (i.e., submovement structure) of one- and two-joint pointing movements by the dominant and non-dominant arms in young and old adults. Based on previous studies [21], we expected to observe differences in the control strategies for the dominant and non-dominant arms. Specifically, it was hypothesized that the dominant arm would be more proficient at utilizing open-loop control (i.e., production of primary submovements that end closer to the target). Furthermore, it was expected that older adults would have more difficulty meeting the accuracy demands of the task thus necessitating a greater incidence of secondary submovements and have primary submovements that end further from the target, especially for movements requiring multi-joint control.

## 2. Materials and Methods

### 2.1. Participants

A total of 12 young (M ± SD: 22 ± 2 years, 7M and 5F) and 12 older (72 ± 8 years, 4M and 8F) participants were recruited to participate in the study. All participants reported being moderately active, having no known neuromuscular disorders, and not taking any medications known to influence neuromuscular performance. The Institutional Review Board at Arizona State University approved the experimental procedures (0407001894, 07/2004) and the participants provided written consent after receiving a written and verbal description of the protocol. All participants indicated right-hand preference based on scores from the Edinburgh Handedness Inventory [36].

### 2.2. Experimental Arrangement

**Figure 1:**
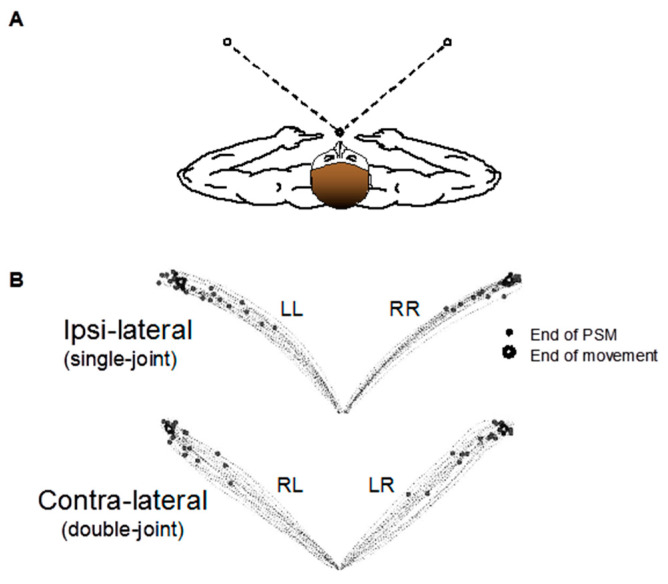
Experimental setup and representative data. (**A**) Participants performed spatially constrained discrete pointing movements to targets situated on either side of their midline using either their right or left arms. (**B**) Experimental data from one older participant. Endpoint (fingertip) trajectories and primary submovement end for each movement condition from a 68-year-old woman. LL: left hand to left target, RR: right hand to right target, RL: right hand to left target, LR: left hand to right target.

Each participant sat upright in an adjustable chair with the torso secured with restraints. The chair was placed closely against a tabletop at approximately the height of the chest. The arms were positioned so that the upper and lower segments were parallel to the floor. The wrist and index finger were constrained from movement by use of a plastic orthosis. The participants’ upper arms were suspended by a sling to eliminate the need for the shoulder muscles to compensate for gravity. Horizontal discrete pointing movements were produced by moving the index finger from a starting position to a target. The starting position for the pointing task was 10 cm in front of the participant’s midline of the body. Circular targets (13 mm in diameter) were drawn on a plexiglass sheet on the tabletop in a position that required 40 degrees of elbow extension on the ipsilateral side to achieve (Figure 1A).

Movement kinematics were recorded with the use of an OPTOTRAK 3-D movement recording system (Northern Digital Inc., Waterloo, ON, Canada) with a sampling frequency of 100 samples/s. An infrared-light emitting diode marker was attached to the distal end of the index fingernail and tracked by the movement recording system to obtain the endpoint kinematics reported here. The first, second, and third derivatives of the endpoint displacement was calculated to obtain the velocity, acceleration, and jerk measures, respectively, that were used in the subsequent determination and analysis of secondary submovement incidence (for an example of the location of the start of secondary submovements in a representative older participant, see Figure 1B).

### 2.3. Experimental Procedures

Prior to being seated at the experimental table, all participants completed the Edinburgh Handedness Inventory to measure the extent of hand preference [36]. The degree of hand preference was quantified by calculating a laterality quotient (LQ) which was based on answers to a 10-point questionnaire. The questionnaire required that a participant indicates which hand would be used for a particular everyday task (e.g., use of a toothbrush). The Purdue Pegboard Test (Lafayette Instrument, Lafayette, IN, USA) was used to characterize differences in the fine motor functional abilities of the young and older participants as well as the dominant and non-dominant arms. Participants had 30 s to place as many small pins in a line of holes as possible using either their left or right arm.

The participants were then positioned at the experimental table where they received a visual demonstration of the pointing task and then performed a minimum number (<5) of familiarization trials to allow them to become familiar with the timing and movement amplitude requirements of the task. Participants performed spatially constrained discrete pointing movements with either the left or right arm from the starting position to the off-center targets. These movements required either elbow extension (ipsilateral movement) or a combination of elbow extension and horizontal shoulder flexion (contralateral movement). In total, there were 4 conditions tested (2 limbs × 2 targets), and participants performed 20 trials in each condition for a total of 80 trials. The order of the conditions was counterbalanced across participants. Participants were instructed to move as “quickly and accurately as possible” to the targets once a starting cue was presented and to keep moving until they had their finger positioned directly on the target. The participants leisurely returned to the starting position after a period of about 3 s from the end of the movement.

### 2.4. Data Analysis

The kinematic data were processed and analyzed in MATLAB 6.5 (MathWorks, Natick, MA, USA) by a method previously described in detail [17,37]. Briefly, the endpoint positional data were dual-passed filtered (2nd order Butterworth, low-pass, 7 Hz). Tangential velocity, acceleration, and jerk were derived and reprocessed using the same filter to minimize noise from the differentiation procedure. The beginning of the movements was defined as the instant when the fingertip velocity exceeded 5% of the peak velocity and proceeded by at least 150 ms where the velocity remained below that threshold for >150 ms. 

The reduction in movements into their primary and secondary submovement phases was achieved by a method described by Meyer and colleagues [16] using an algorithm reported by Novak and colleagues [38,39] where the time period between peak velocity and the end of the movement was examined for one of two events: (1) a negative to positive zero crossing in acceleration, (2) a positive to negative zero crossing in jerk. That instant was then designated as the end of the primary submovement. However, if the end of the primary submovement coincided with the end of the movement, no secondary submovements were registered.

### 2.5. Statistical Analysis

The Edinburgh Handedness Inventory data were analyzed with an independent-samples *t*-test to determine if the groups differed on strength of hand preference. Purdue pegboard test performance was analyzed using a 2 × 2 ANOVA (young vs. older; right vs. left arm). For the pointing task, a mixed three factor ANOVA (2 age × 2 arm × 2 targets) was used to analyze the dependent variables (i.e., movement time, incidence of secondary submovements, and duration of primary submovements) for the existence of statistical main effects and interactions on arm used (right vs. left), on target position (ipsilateral vs. contralateral), and on age (young vs. older). Post hoc analyses using Bonferroni adjustments were performed as necessary. All alpha levels were set at *p* < 0.05 and statistical analyses were implemented with SPSS 14.0 (SPSS Inc., Chicago, IL, USA). The data are reported as means ± S.D. in the text and presented as means ± S.E. in the figures.

## 3. Results

### 3.1. Participant’s Perferred Arm Characterization

All participants reported the preferred use of the right arm and hand for writing. Furthermore, based upon the LQ measured from the Edinburgh Handedness Inventory, there were no differences between young and older adults in the extent of hand preference (Table 1; *p* = 0.7). These relatively high LQ values indicated that both groups were comprised of individuals with a high degree of right-hand preference (i.e., strong right handers; LQ = 0.82 ± 0.19, range 0.2 to 1.0).

The Purdue pegboard test was administered to determine functional differences between the left and right arms in young and old adults. Young participants performed better than older participants for both the right (14.9 ± 1.5 vs. 13.0 ± 2.2 pins) and the left arms (13.8 ± 2.0 vs. 11.7 ± 2.3 pins; *p* < 0.001). Furthermore, there were within-group differences between the left and right arms with the right arm performance exceeding that of the left arm for both young (14.9 ± 1.5 vs. 13.8 ± 2.0; *p* < 0.05) and old (13.0 ± 2.2 vs. 11.7 ± 2.3; *p* < 0.05) (Table 1).

### 3.2. Movement Time and Speed

**Figure 2:**
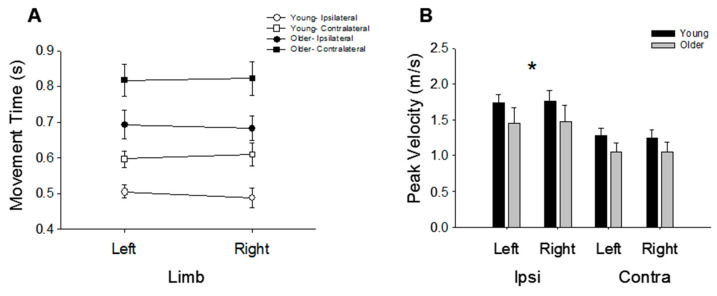
Movement time and movement speed. (**A**) Movement time was greater for older adults (*p* < 0.001) and contralateral movements (*p* < 0.001). There were no differences between the right and left hands. (**B**) Peak velocity was similar between hands for the ipsilateral and contralateral movements and for the young and older adults. However, the contralateral movements were characterized by a lower peak velocity compared with the ipsilateral movements (* *p* < 0.001).

In both young and older adults, the two-joint contralateral movements were slower than the one-joint ipsilateral movements (0.70 ± 0.18 vs. 0.58 ± 0.16 ms; *p* < 0.01; Figure 2). In addition, older participants demonstrated slower movement times compared with younger participants for both the ipsilateral movements (0.69 ± 0.13 vs. 0.47 ± 0.09 ms; *p* < 0.001) and the contralateral movements (0.82 ± 0.16 vs. 0.59 ± 0.10 ms; *p* < 0.001). There were no differences between movement times of the right and left arms for either the young (Contra: *p* = 0.55; Ipsi: *p* = 0.24) or older participants (Contra: *p* = 0.78; Ipsi: *p* = 0.56; Figure 2A).

Figure 2B shows an average peak velocity for each movement condition in young and older participants. There were no differences in peak velocity between the young and older participants (*p* = 0.24). For both age groups combined, the ipsilateral movements displayed higher peak velocities compared with the contralateral movements for both the left (1.59 ± 0.60 vs. 1.17 ± 0.39 m/s; *p* < 0.001) and right arms (1.61 ± 0.69 vs. 1.15 ± 0.40 m/s; *p* < 0.001). However, there were no differences between arms for the ipsilateral (*p* = 0.59) or contralateral movements (*p* = 0.56).

### 3.3. Submovement Analysis

**Figure 3:**
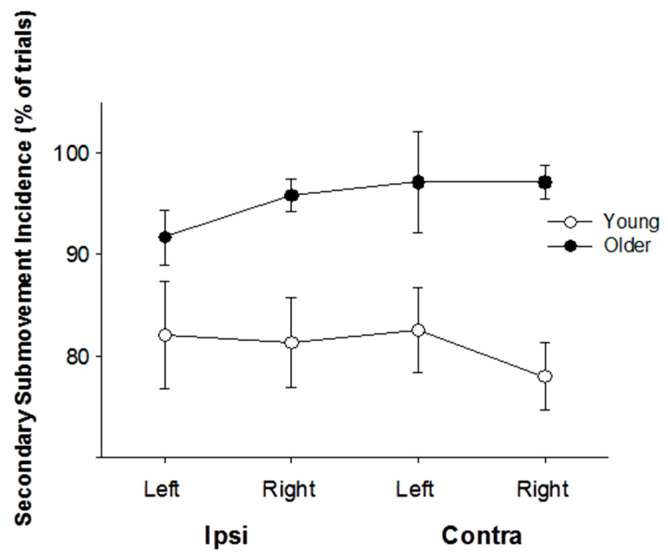
Older adults produce secondary submovements more often than young adults. This figure shows the percentage of trials in which the target was not achieved with the primary submovement and required a secondary submovement. In all conditions, young participants needed a secondary submovement in fewer trials compared with the older group (*p* < 0.01).

Older participants produced secondary submovements more often than the young participants. Figure 3 illustrates the percentage of trials in which the target was not achieved with the primary submovements and thus required a secondary submovement. For all movement conditions (i.e., left and right arm, ipsilateral and contralateral), 81 ± 14% of trials for the young participants required secondary submovements, whereas older participants more often required secondary submovements (93 ± 6% of trials; *p* < 0.01). There were no differences between hands (*p* = 0.14), or ipsilateral and contralateral movements (*p* = 0.63).

**Figure 4:**
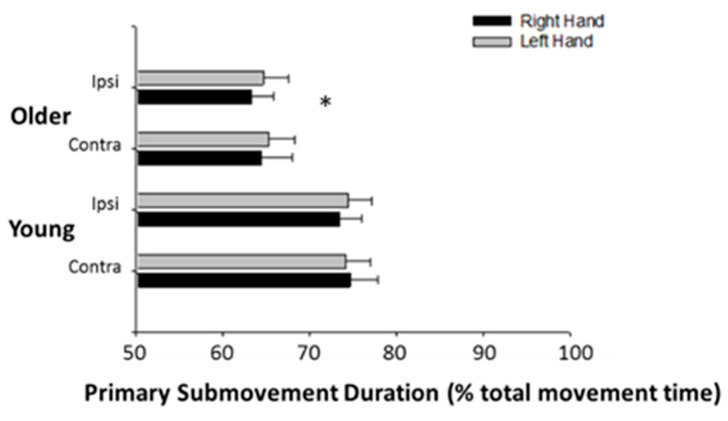
Primary submovements are relatively shorter in older adults. Primary submovements duration normalized by total movement time was shorter for older adults (* *p* < 0.01). There were no differences between hands or ipsilateral and contralateral movements.

In absolute terms, the primary submovements were longer in older participants. However, since there were differences between the young and older groups in movement time, the absolute duration of the primary submovement was normalized by total movement time for that particular trial. This revealed that primary submovements were relatively longer in duration in young participants, meaning they spent more of the total movement time performing the primary submovement (Figure 4) (young: 74.2 ± 9.9%, older: 64.4 ± 10.2%; *p* < 0.01). Conversely, older participants had relatively shorter primary submovement durations, meaning they spent less time in the primary submovement and more time in the corrective secondary submovement phase. There were no differences in the normalized duration of the primary submovements for the ipsilateral or contralateral movements in the left and right arms in either the young or older participants (*p* = 0.61).

## 4. Discussion

The purpose of the study was to determine the influence of limb preference on discrete movements requiring spatial accuracy in young and older adults. Specifically, we examined the kinematic structure of elbow extension pointing movements for the incidence of submovements and duration of primary submovements to infer differences in open-loop and feedback control of the limbs. There were three main findings: (1) there were no interlimb (i.e., left vs. right) differences or (2) differences in either the ipsilateral or contralateral (i.e., one-and two-joint) movements in the incidence or relative duration of primary submovements for either the young or older participants. Furthermore, (3) there were age-related differences in the incidence and relative duration of primary submovements.

### 4.1. Arm Preference and Performance

The seminal doctoral studies of Robert S. Woodworth were the first to experimentally examine and document accuracy differences between the left and right arms. Subsequently, investigators have studied the motor asymmetries and have sought to identify the physiological mechanisms responsible for those performance differences. Following along the lines of Paul Broca’s and Carl Wernicke’s discovery of the specialization of the left-cerebral hemisphere for language, the performance differences in the left and right limbs are mostly attributed to differences in cerebral hemispheric processing [40] with the left hemisphere (i.e., right hand) often demonstrating superior movement control. More recently, Sainburg and colleagues have expanded this explanation of hemispheric differences in the control of the dominant and non-dominant limbs with the formulation of the dynamic-dominance hypothesis of handedness. This hypothesis is based upon the observation that the nervous system processes controlling the non-dominant arm appear to be specialized for static position control and those controlling the dominant arm specialized for trajectory control [8,9]. The hypothesis would have predicted that for the current study the dominant arm should have performed better (i.e., lower incidence of secondary submovements and longer primary submovements) or at least differently than the non-dominant arm. The lack of differences between the limbs in the submovement structure analysis, at least in with the current study task conditions, did not lend support to the dynamic-dominance hypothesis. However, it still appears clear that the limbs are specialized for other aspects of control and function [7,9,23].

Movement times in the present study were not affected by the arm used for either the ipsilateral or contralateral movements. This was not expected as it is generally accepted that the right arm exhibits quicker movement times [5,24], although this is not always the case [41]. However, the ipsilateral movements were performed faster than the contralateral movements although the target size and distance from the starting position to the targets were identical. This has been shown before and is attributed to inertial resistance the arm experiences while moving across the body [42] and an advantage to arms moving in their own hemispace [43]. There were no inter-limb differences in the submovement analysis of pointing movements in either young or older adults for the ipsilateral and contralateral movements performed in the current study. The limbs produced a similar number of trials in which secondary submovements were required and exhibited primary submovements of similar durations. Based upon assumptions made in the stochastic optimized-submovement model [16], this would indicate that both limbs utilized open-loop and feedback control to similar extents. Therefore, the findings were not consistent with differential feed-forward/feedback control of the right and left arms as suggested by some authors [21,23,25,44]. Others have questioned the origin of the submovements [17,45] as well as the dichotomous distinction of open-loop and feedback control of the upper limbs [8,9].

It is likely that the constraints (one- and two-joint movements) imposed on our reaching movements in the current study influenced our findings. In the interpretation of our results, it is important to distinguish between skilled and independent movements. Skilled movements are performed during everyday tasks such as handwriting, and in relatively complex evaluative tasks such as the Purdue pegboard test. These types of movements require fine control and coordination of individual synergist and antagonist muscles. In contrast, independent movements require isolation of the muscles controlling the movement, which are usually restricted to experimental examinations such as the current study. Although we utilized a task with varying levels of difficulty (i.e., horizontal one-joint and two-joint movements), these movements require relatively little skill and motor control. It is in those skilled tasks where significant performance advantages are usually observed with the dominant hand [5]. The differences found between the limbs for pin placement in the Purdue pegboard test, but not for the pointing task support this view. In addition, a stronger transport-grasp linkage has been found in the dominant hand [46], which would likely contribute to the bilateral performance differences in a task such as the Purdue pegboard test.

### 4.2. The Effects of Age on Performance

As a result of the normal aging process, older adults often exhibit slower and less accurate movements [26,47,48]. The results of the current study are in line with this observation. Specifically, the older adults in our study had longer movement times, displayed secondary submovements more often, and displayed primary submovements that ended further from the target compared with young adults. In combination, these measures in older adults demonstrate the greater difficulty in producing accurate pointing motions (i.e., achieving the target with the primary submovement). Additionally, the similar peak velocities recorded in the young and older adults indicates that the movement slowing was related to a change in the submovement structure and not necessarily to a physiological phenomenon such as a decline in conduction velocity of neurons or reductions in muscle mass. An alternative interpretation proposes that the kinematic fluctuations recorded as submovements are a direct result of slower movement in older adults [32]. However, the traditional interpretation of the origin of the submovements (i.e., used for corrective adjustments) suggests that open-loop control for rapid aiming tasks is compromised in older adults necessitating a greater reliance on feedback control closed-loop processes [31,49,50,51] and causing movement slowing to acquire the target [52].

The origin of these commonly observed open-loop accuracy deficits in older adults have been explained by age-related deficits in strength and central planning [49,51]. An additional possible contributor involves the increase in force fluctuations expressed by older adults while attempting to perform steady contractions at lower force levels [53,54], which itself has been ascribed to age-related changes in the properties of individual and population activation of motor units [55,56,57,58]. The increased force fluctuations work to influence the ability to produce a smooth trajectory and ultimately contribute to the capacity to acquire the desired target location [59,60]. Additionally, the motor abilities in the arms of older adults are known to be further compromised by an altered neural strategy which has been shown to include altered activation of agonist muscles [61] and heightened activation of antagonist muscles [33,62,63], although these changes may serve to dampen the force fluctuations and improve performance [33,54].

Right-handed older adults often exhibit a stronger preference to utilize their dominant limbs compared with younger adults in most everyday tasks [64,65]. The shift to stronger right-hand preference is the basis of the right-hemisphere aging model which proposes that the right cerebral hemisphere (and thus left hand control) is affected by aging processes to a greater extent than the left hemisphere [66,67]. The results obtained from the Edinburgh Handedness Inventory in the current study showed that the strength of hand preference for this sample of young and older adults was similar (Table 1). Furthermore, there were no differences between the performance of the right or left hands in older participants for submovement incidence. Therefore, at least for simple aiming tasks, the notion of asymmetrical cerebral hemispheric aging has not been supported.

## 5. Conclusions

No interlimb differences in the movement structure suggests that both arms utilized similar open-loop control during the simple elbow extension pointing movements. Furthermore, the age-related differences in the submovement structure suggests compromised open-loop control in older adults which contributes to movement slowness.

## Figures and Tables

**Table 1:** Participant handedness characterization. Data represent means ± S.D. for age and hand preference. * *p* < 0.05 compared with left hand. † *p* < 0.001 compared with older adults.

			Purdue Pegboard Test (Pegs/30 s)	
	Age (Years)	Laterality Quotient	Right Hand	Left Hand
Young (*n* = 12)	22 ± 2	0.81 ± 0.21	14.9 ± 1.5 * †	13.8 ± 2.0 †
Older (*n* = 12)	72 ± 8	0.83 ± 0.18	13.0 ± 2.2 *	11.7 ± 2.3

## Data Availability

The data presented in this study are available on request from the corresponding author.
